# The Interpersonal Circumplex and the Alternative Model of Personality Disorders: Relationships among Older Adults

**DOI:** 10.1093/geroni/igab046.3241

**Published:** 2021-12-17

**Authors:** Lisa Stone, Daniel Segal

**Affiliations:** 1 University of Colorado Colorado Springs, Colorado Springs, Colorado, United States; 2 University of Colorado at Colorado Springs, University of Colorado at Colorado Springs, Colorado, United States

## Abstract

Introduction: The interpersonal circumplex model measures interpersonal dysfunction along two axes (communion and agency), resulting in eight unhealthy patterns: Domineering, Vindictive, Cold, Socially Avoidant, Nonassertive, Exploitable, Overly Nurturant, and Intrusive. It is unclear how the circumplex model applies to older adults and their unique biopsychosocial contexts. This study examined relationships between the circumplex and personality disorder features, using the Alternative Model of Personality Disorder’s (AMPD) personality functioning and pathological personality trait constructs. Method: Older adults (N = 202) completed the Inventory of Interpersonal Problems-Short Circumplex (IIP-SC), the Levels of Personality Functioning Scale-Self-Report (LPFS-SR), and the Personality Inventory for DSM-5 (PID-5) to measure pathological personality traits. Results: Correlations were computed between the IIP-SC’s eight circumplex scales with the LPFS-SR’s four personality functioning domains and with the PID-5’s five domains. All circumplex scales significantly (p < .001) and positively correlated with all LPFS-SR and PID-5 domains, with large effect sizes (> .45). Next, regressions were conducted, with the LPFS-SR and PID-5 domains predicting each IIP-SC scale. Across the eight regressions, the AMPD constructs accounted for significant variance in the IIP-SC scales, ranging from 38% (Nonassertive) to 64% (Domineering and Cold). Discussion: Significant overlap between the interpersonal circumplex and the AMPD was demonstrated, but patterns are distinct from previous research among younger adults. The circumplex was limited in its relation to the AMPD’s personality functioning, but the pathological personality trait model was well represented through the circumplex. Results indicate that the circumplex may have some validity among older adults and warrants further investigation.

